# *APOE* ε4 Is Associated with Disproportionate Progressive Hippocampal Atrophy in AD

**DOI:** 10.1371/journal.pone.0097608

**Published:** 2014-05-30

**Authors:** Emily N. Manning, Josephine Barnes, David M. Cash, Jonathan W. Bartlett, Kelvin K. Leung, Sebastien Ourselin, Nick C. Fox, for the Alzheimer's Disease NeuroImaging Initiative

**Affiliations:** 1 Dementia Research Centre, UCL Institute of Neurology, London, United Kingdom; 2 Centre for Medical Image Computing, University College London, London, United Kingdom; 3 Department of Medical Statistics, London School of Hygiene and Tropical Medicine, London, United Kingdom; Univ. Kentucky, United States of America

## Abstract

**Objectives:**

To investigate whether *APOE* ε4 carriers have higher hippocampal atrophy rates than non-carriers in Alzheimer's disease (AD), mild cognitive impairment (MCI) and controls, and if so, whether higher hippocampal atrophy rates are still observed after adjusting for concurrent whole-brain atrophy rates.

**Methods:**

MRI scans from all available visits in ADNI (148 AD, 307 MCI, 167 controls) were used. MCI subjects were divided into “progressors” (MCI-P) if diagnosed with AD within 36 months or “stable” (MCI-S) if a diagnosis of MCI was maintained. A joint multi-level mixed-effect linear regression model was used to analyse the effect of ε4 carrier-status on hippocampal and whole-brain atrophy rates, adjusting for age, gender, MMSE and brain-to-intracranial volume ratio. The difference in hippocampal rates between ε4 carriers and non-carriers after adjustment for concurrent whole-brain atrophy rate was then calculated.

**Results:**

Mean adjusted hippocampal atrophy rates in ε4 carriers were significantly higher in AD, MCI-P and MCI-S (p≤0.011, all tests) compared with ε4 non-carriers. After adjustment for whole-brain atrophy rate, the difference in mean adjusted hippocampal atrophy rate between ε4 carriers and non-carriers was reduced but remained statistically significant in AD and MCI-P.

**Conclusions:**

These results suggest that the *APOE* ε4 allele drives atrophy to the medial-temporal lobe region in AD.

## Introduction

Hippocampal atrophy rate has been proposed as an imaging biomarker for Alzheimer's disease (AD) progression [1], [2]. However, it is essential to understand how factors might affect hippocampal atrophy rates if this biomarker is to be used most effectively in clinical trials.

Arguably, the most important genetic risk factor for sporadic AD is the ε4 variant of the *APOE* gene [3]. Of the three common alleles of the *APOE* gene, ε3 is most frequent with ε4 less common and ε2 relatively rare [4]. ε4 increases the risk of AD and lowers the age of disease onset [5]. There is also evidence that the topography of atrophy in ε4 carriers (ε4+) may be different from non-carriers (ε4-) in AD [6]–[9] although not all studies have confirmed this [10].

Numerous publications have attempted to elucidate whether *APOE* modifies hippocampal atrophy rates [11]–[25]. Although some studies reported elevated hippocampal atrophy rates in ε4+ in AD, mild cognitive impairment (MCI) and control groups, it is possible that the greater hippocampal rates observed could have been attributed to higher concurrent whole-brain atrophy rates and therefore faster disease progression.

To better understand the effect of the *APOE* ε4 allele on the progression of structural brain changes we wanted to investigate whether different whole-brain and hippocampal atrophy rates were observed in ε4+ compared with ε4- in AD, MCI and controls. Further, we wanted to investigate if there is evidence of higher hippocampal atrophy rates in ε4+ when adjusting for concurrent whole-brain atrophy rates, which to our knowledge, has not been examined.

## Methods

### Ethics Statement

Data used in preparation of this article were obtained from the Alzheimer's Disease Neuroimaging Initiative (ADNI) database (adni.loni.ucla.edu). ADNI is a multi-centre study with data collected from over 50 sites. The institutional review board at all participating sites approved the study and written consent was obtained from all participants. More information can be found at http://www.adni-info.org/scientists/Pdfs/ADNI_Protocol_Extension_A2_091908.pdf.

### Subjects

ADNI is a multi-centre public/private funded longitudinal study investigating adult subjects with AD, amnestic MCI, and normal cognition. Participants underwent baseline and periodically repeated clinical and neuropsychometric assessments and MRI. Subjects from ADNI who had a baseline MRI scan and at least 1 follow-up scan were included in this study. Each subject underwent *APOE* genotyping at the screening visit. Detailed inclusion criteria for the ADNI study can be found at http://www.adni-info.org/scientists/Pdfs/ADNI_Protocol_Extension_A2_091908.pdf. All demographic information, diagnoses, neuropsychological test scores and *APOE* genotype data were downloaded from the ADNI clinical data repository.

**Figure 1:**
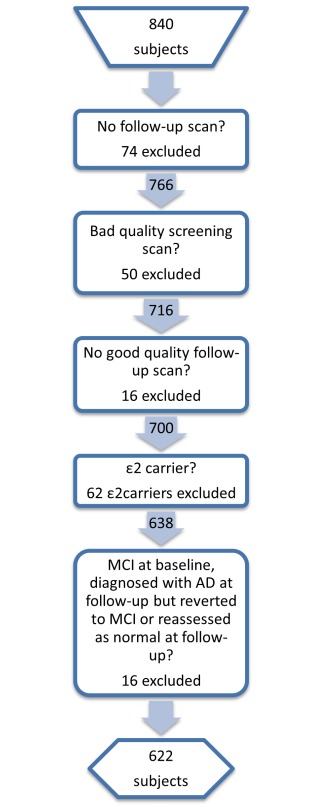
Subject selection process.

Since a proportion of MCI subjects will likely not progress to dementia caused by AD, this group is likely to be quite heterogeneous with respect to underlying pathology. As a result, we dichotomised the MCI subjects into those who were observed to progress to a clinical diagnosis of AD within 36 months of baseline and maintained that diagnosis (MCI-P) and those who were stable over the follow-up period (MCI-S). Subjects whose diagnosis changed from MCI to AD and subsequently reverted to MCI during the study were excluded as were subjects whose diagnosis changed from MCI to normal. ε2 carriers (i.e. ε2/ε2, ε2/ε3 and ε2/ε4 subjects) were also excluded from the study as they may have lower hippocampal atrophy rates [26]. There were a total of 840 ADNI subjects available at the time of this study, after exclusions this number reduced to 622 subjects. The number of subjects excluded at each exclusion stage is summarised in Figure 1.

### Image acquisition and analysis

The ADNI MRI protocol used in this study is described elsewhere [27]. Two T1-weighted MRI scans (MPRAGE) were acquired at each session. The higher quality image (as assessed by a single quality control centre) was selected. Pre-processing corrections were then applied depending on the scanner manufacturer and head coil used: 1) correction for image geometry distortion due to gradient non-linearity (gradwarp) [28], 2) B1 non-uniformity correction [29] and 3) intensity non-uniformity correction (N3 histogram peak sharpening)[30]. After pre-processing, the scans were additionally visually inspected at the Dementia Research Centre for motion artefacts. Those scans with significant motion artefacts were excluded from the current study. Whole-brain and hippocampi were automatically delineated using the Multi-Atlas Propagation and Segmentation technique (MAPS) from the pre-processed 1.5-T T1-weighted MRI scans at all available time-points [31], [32]. The whole-brain MAPS technique uses a template library of semi-automatically segmented whole-brain regions (comprised of grey and white matter containing voxels with the brain-stem included up until the most inferior slice containing cerebellum) and the hippocampal MAPS technique uses a template library of manually segmented hippocampal regions. The MAPS technique works by comparing the target image to these templates and the best-matched templates are then combined to generate the segmentation of the target image. The change in the volumes of the whole-brain and hippocampi between follow-up and baseline were calculated using the robust boundary shift integral (KN-BSI) [33]. Total intracranial volume (TIV) was estimated by summing the volumes of grey matter, white matter, and cerebrospinal fluid (CSF) segmentations using SPM8 (http://www.fil.ion.ucl.ac.uk/spm/software/spm8). Brain-to-TIV ratio was calculated by dividing the extracted whole-brain volumes by the extracted TIVs. A list of the subjects and time points included in the analysis can be found in appendix S1.

### Statistical Analysis

All statistical analyses were performed in Stata (version 12). *APOE* ε4 carrier status was coded as 1 for carriers of 1 or 2 ε4 alleles and 0 for those who did not carry an ε4 allele. We analysed the effect of *APOE* ε4 carrier-status on the volume of the sum of the left and right hippocampi at baseline adjusting for the level of overall whole-brain atrophy. To do this a linear regression was performed within each clinical group with bilateral hippocampal volume as the dependent variable and *APOE* ε4 carrier-status, age, gender, MMSE score, TIV and brain-to-TIV ratio included as covariates. Age was included as a covariate as normal aging is associated with brain volume loss, TIV to control for variation in head size and gender to control for any differences in male-to-female ratio between the different genotype groups. We included MMSE score and brain-to-TIV ratio as covariates in order to assess the effect of the *APOE* ε4 carrier-status above and beyond any global differences in cognitive impairment and whole-brain atrophy.

To analyse the effect of the *APOE* ε4 carrier-status on the rate of atrophy of the hippocampi and whole-brain (as measured using the BSI), joint linear mixed models were used. These models allow the random-effects dictating the trajectories of hippocampal and whole-brain atrophy to be correlated, thus permitting estimates of hippocampal atrophy rate adjusted for true whole-brain atrophy rate. They allow for repeated measures and accommodate missing values under the missing at random assumption. The dependent variables were the ml loss of hippocampi as calculated by the hippocampal-BSI and brain as calculated by the brain-BSI.

Interval (years) between baseline and follow-up scans was included as a fixed-effect and interactions terms between *APOE* ε4 carrier-status and scan interval were included to allow hippocampal atrophy rate to vary with *APOE* ε4 carrier-status. Interactions of interval with age, MMSE score, brain-to-TIV ratio, gender and TIV (all measured at baseline) were also included as fixed-effects in the model. Interval was also included as a random-effect, to allow for between subject heterogeneity in atrophy rate. No constant terms (fixed or random) were included, consistent with the assumption that true (as opposed to measured) atrophy between two scans from the same time-point is zero. A single joint model was fitted to both hippocampal and whole brain losses, allowing distinct fixed and random effect parameters for the two processes. The two trajectories were linked through a correlation between the two random slopes. The difference in mean hippocampal rates between ε4+ and ε4− after adjustment for concurrent brain atrophy rate was then estimated. This was calculated as the difference in hippocampal rates (unadjusted for brain atrophy rate), minus the difference attributable due to differences in brain rates (based on the standard deviations of the random-slopes and their correlation in the joint model). See appendix s2 for the expressions of the statistical models used.

Since we included gender as a binary categorical variable in our analyses we chose to present mean adjusted values for a 50/50 split of males: females in the Figures and Tables (adjusted for disease-group specific mean age, baseline brain-to-total intracranial volume ratio, MMSE score and total intracranial volume). The mean adjusted values for a 50/50 gender split were calculated by multiplying the coefficients for males and females by 0.5 and adding them together. Given that we did not include an interaction term between ε4 carrier-status and gender in our analyses, the differences in whole-brain and hippocampal atrophy rates are the same for males and females.

## Results

Table 1 shows demographics and imaging summary statistics for each clinical group used in this study. As previously shown [31], the AD subjects had smaller mean hippocampal volumes at baseline than MCI subjects whose hippocampi were in turn smaller than control subjects (Table 1); the mean hippocampal volume for the AD subjects was ∼20% smaller than the controls with the MCI-P and MCI-S subjects having intermediate volumes.

**Table 1:** Baseline demographics and image summary statistics by clinical group.

	Controls	MCI stable	MCI progressors	AD
No. Subjects (at 6 m, at 12 m, at 18 m, at 24 m, at 36 m)	167 (165, 153, 0, 137, 115)	169 (157, 147, 125, 103, 66)	138 (133, 131, 116, 102, 69)	148 (143, 124, 1, 93, 1)
No. ε4 non-carriers (% total), No. ε4 heterozygotes (% total), No. ε4 homozygotes (% total)	118 (71%), 44 (26%), 5 (3%)	86 (51%), 68 (40%), 15 (9%)	42 (30%), 70 (51%), 26 (19%)	44 (30%), 70 (47%), 34 (23%)
% male	54%	66%	59%	55%
Age [years]	76.0 (5.1)	75.5 (7.2)	74.2 (6.9)	75.0 (7.6)
MMSE score	29.2 (0.9)	27.2 (1.8)	26.6 (1.7)	23.4 (1.9)
TIV [cm3]	1548 (143)	1558 (142)	1552 (156)	1537 (167)
Unadjusted mean bilateral baseline hippocampal volume [cm^3^]	5.2 (0.7)	4.6 (0.8)	4.2 (0.8)	3.9 (0.9)

Age, TIV, MMSE and unadjusted hippocampal volume (left and right summed) are given as mean (SD).

### Baseline cross-sectional results

**Figure 2:**
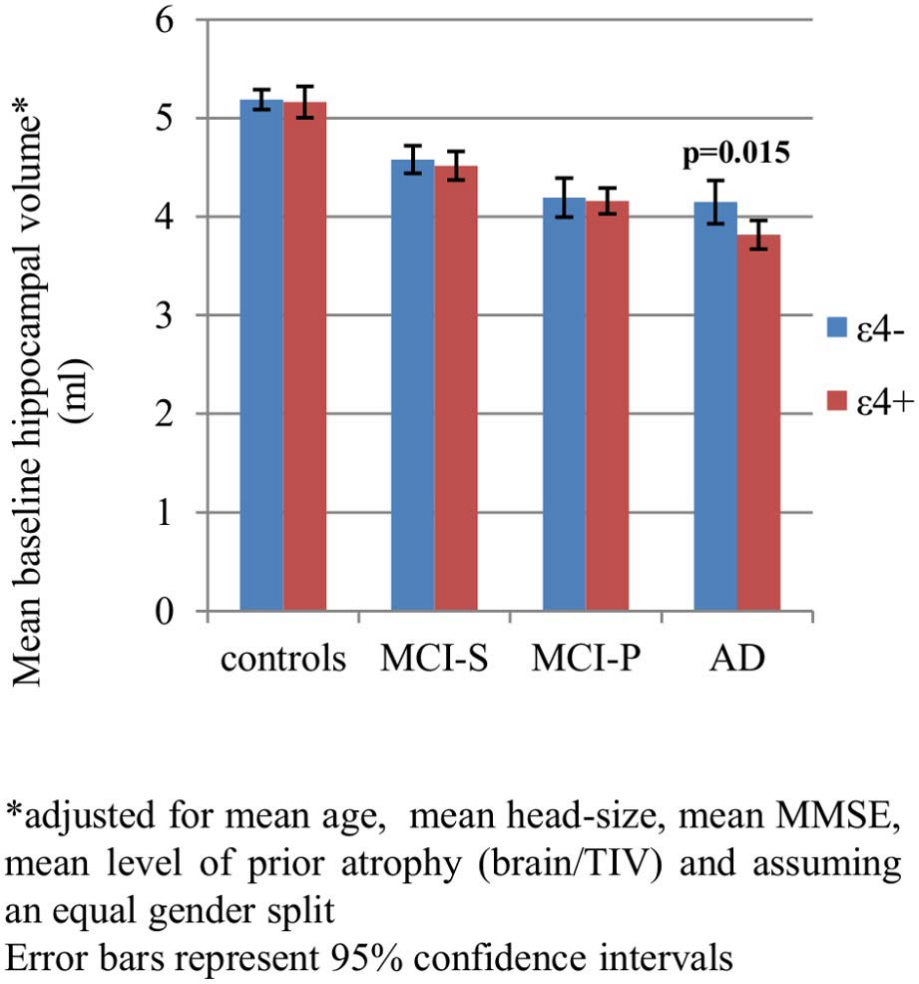
Effect of *APOE* ε4 on baseline hippocampal volumes.*

Table 2 and Figure 2 show the results of the cross-sectional analysis of hippocampal volumes. In AD, after adjustment for age, gender, MMSE score, brain-to-TIV ratio and TIV, the mean baseline hippocampal volume of ε4+ was significantly smaller than that of ε4- (by ∼8%). There was no evidence of a difference in mean adjusted baseline hippocampal volume between ε4 carriers and non-carriers in MCI-P, MCI-S or controls.

**Table 2:** Adjusted mean baseline hippocampal volumes for ε4 non-carriers and adjusted mean differences in total (left and right summed) baseline hippocampal volumes between ε4 carriers and non-carriers in controls, stable MCI, MCI progressors and AD (-ve sign means ε4+ < ε4-).

	Controls (ε4- = 118, ε4+ = 49)	MCI-S (ε4- = 86, ε4+ = 83)	MCI-P (ε4- = 42, ε4+ = 96)	AD (ε4- = 44, ε4+ = 104)
Mean adjusted* baseline hippocampal volume** in ε4- (cm3) [95% CI]	5.19 [5.08, 5.29]	4.58 [4.44, 4.72]	4.19 [4.00, 4.39]	4.15 [3.93, 4.37]
Difference in mean adjusted* baseline hippocampal volume** between ε4+ and ε4- (cm3) [95% CI]	−0.02 [−0.21, 0.16] p = 0.811	−0.06 [−0.26, 0.13] p = 0.508	−0.03 [−0.27, 0.20] p = 0.772	−0.33 [−0.59, −0.07] p = 0.015

* all values are for a 50/50 gender split and are adjusted for disease-group specific mean age, baseline brain-to-total intracranial volume ratio, MMSE score, and total intracranial volume. **average of left and right.

### Longitudinal Results

**Figure 3:**
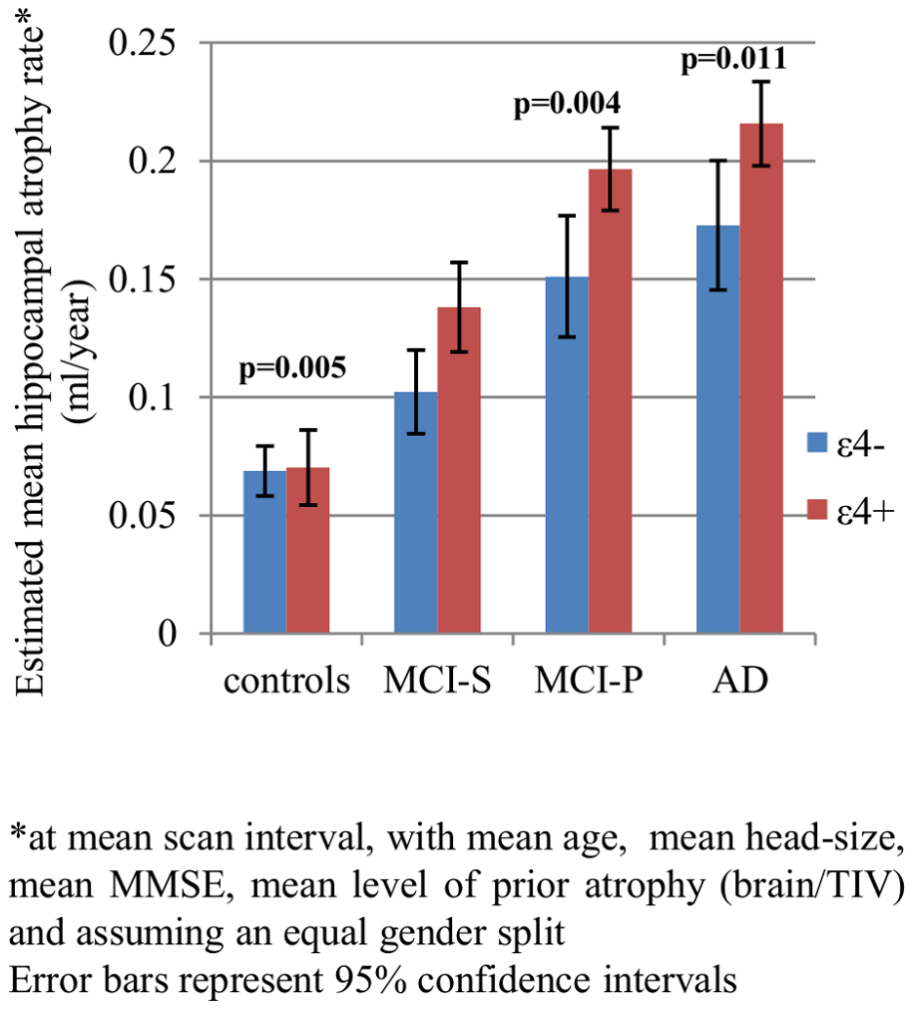
Effect of *APOE* ε4 on hippocampal atrophy rates.*

**Figure 4:**
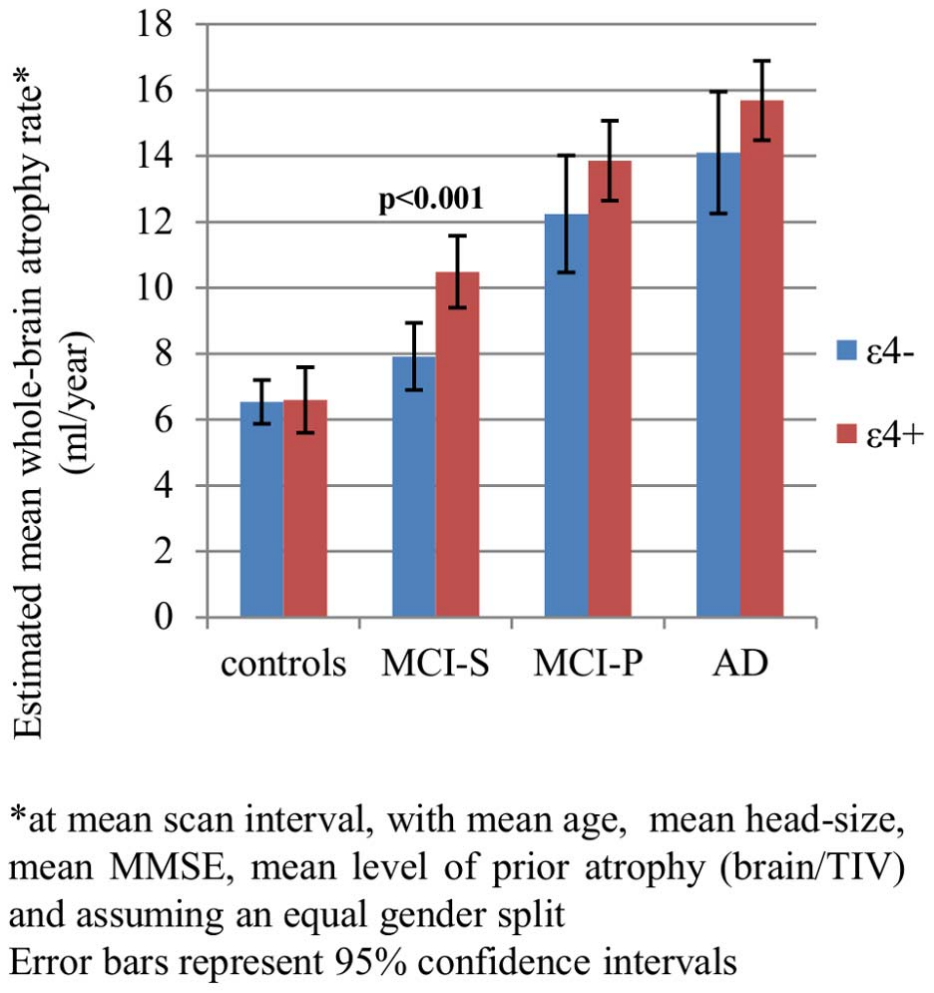
Effect of *APOE* ε4 on whole-brain atrophy rates.*

**Figure 5:**
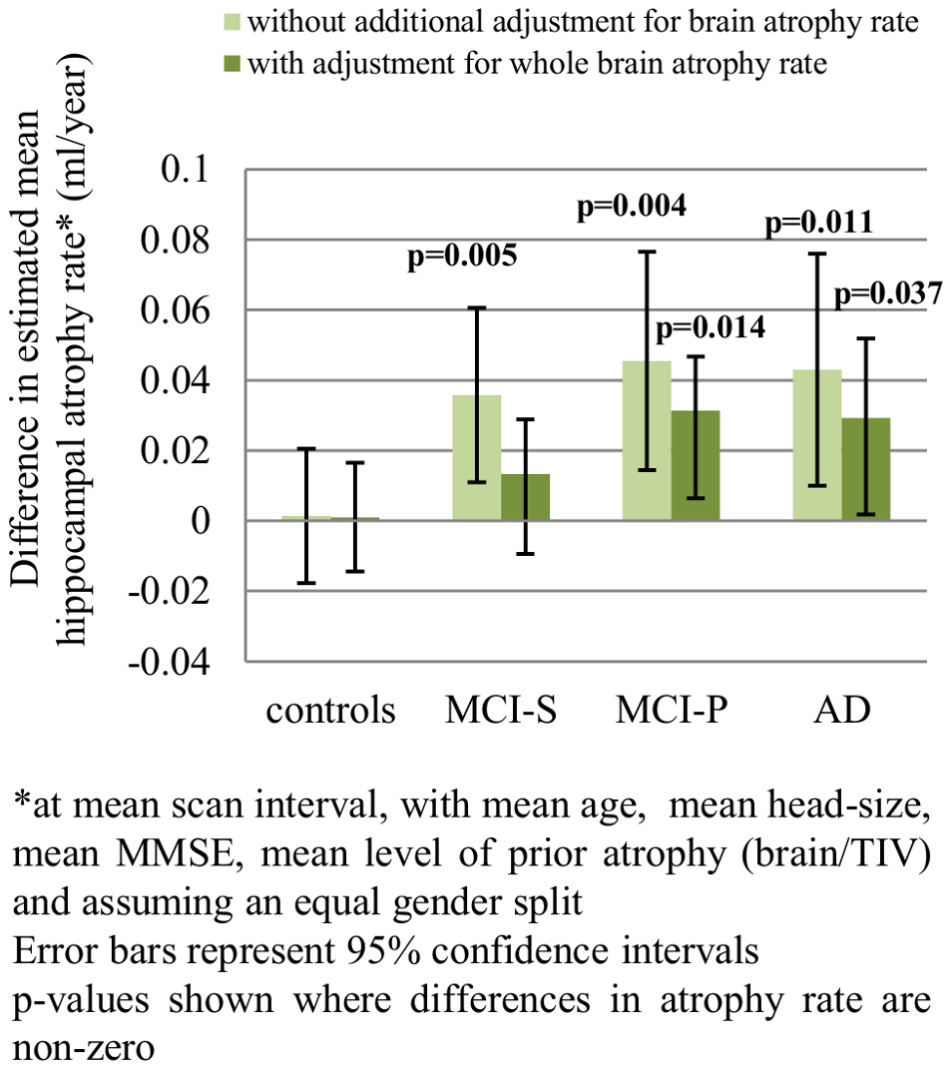
Difference in hippocampal atrophy rates*: ε4+ vs ε4-.

Table 3 and Figure 3, Figure 4 and Figure 5 show the results of the longitudinal analyses of the differences in mean adjusted atrophy rates between ε4+ and ε4- in all subject groups.

**Table 3:** Adjusted mean difference in whole-brain and hippocampal atrophy rate (ml) [95% CI] for ε4 carriers compared with non-carriers in controls, stable MCI, MCI progressors and AD (+ve means atrophy rate is higher in ε4+).

		ε4 carrier status	Controls (ε4− = 118, ε4+ = 49)	MCI stable (ε4− = 86, ε4+ = 83)	MCI progressors (ε4− = 42, ε4+ = 96)	AD (ε4− = 44, ε4+ = 104)
Whole-brain	Mean adjusted* atrophy rate (ml/year)	ε4−	6.54 [5.88, 7.20]	7.91 [6.90, 8.93]	12.24 [10.47, 14.02]	14.11 [12.26, 15.96]
	Difference in mean adjusted* atrophy rate (ml/year)	ε4+ vs ε4−	0.05 [−1.15 1.25] p = 0.938	2.57 [1.14, 4.00] p<0.001	1.62 [−0.54, 3.77] p = 0.142	1.58 [−0.65, 3.81] p = 0.165
Hippocampus**	Mean adjusted* atrophy rate (ml/year)	ε4−	0.069 [0.058, 0.079]	0.102 [0.085, 0.120]	0.151 [0.125, 0.177]	0.173 [0.145, 0.200]
	Difference in mean adjusted* atrophy rate (ml/year)	ε4+ vs ε4−	0.001 [−0.018, 0.021] p = 0.881	0.036 [0.011, 0.061] p = 0.005	0.045 [0.014, 0.076] p = 0.004	0.043 [0.010, 0.076] p = 0.011
	Difference in mean adjusted* atrophy rate after adjustment for concurrent whole-brain atrophy rate (ml/year)	ε4+ vs ε4−	0.001 [−0.014, 0.016] p = 0.897	0.013 [−0.009, 0.036] p = 0.250	0.031 [0.006, 0.056] p = 0.014	0.029 [0.002, 0.057] p = 0.037

* all values are for a 50/50 gender split and are adjusted for disease-group specific mean age, baseline brain-to-total intracranial volume ratio, MMSE score, and total intracranial volume.

**average of left and right.

We found statistically significant evidence that in AD, MCI-P and MCI-S subjects, after adjusting for age, gender, TIV, MMSE score and brain-to-TIV ratio, the mean hippocampal atrophy rates were higher in ε4+ compared with ε4- (see Figure 3). Mean adjusted brain atrophy rates were also higher in ε4+ compared with ε4-, but only significantly so in the MCI-S group (see Figure 4). After adjustment for concurrent whole-brain atrophy, the difference in atrophy rate between ε4+ and ε4-was reduced by ∼ 25% in AD, by ∼40% in MCI-P and by ∼75% in MCI-S (see Figure 5). Although the differences in mean adjusted hippocampal atrophy rates were reduced when additionally adjusting for concurrent whole-brain loss, differences between ε4+ and ε4- remained statistically significant in AD and MCI-P. In the control group there was no evidence that hippocampal or whole-brain atrophy rate differed between ε4+ and ε4- (p>0.8 for both).

## Discussion

This study examined the effect of *APOE* genotype on hippocampal volumes and hippocampal atrophy rates in AD, MCI and in controls, with and without adjusting for concurrent brain atrophy rates.

Cross-sectionally we found evidence that AD ε4+ had smaller (∼8%) mean hippocampal volumes at baseline than ε4- after adjusting for age, TIV, gender, MMSE score and brain-to-TIV ratio. There was no evidence that ε4+ had smaller hippocampal volumes than non-carriers in MCI-P, MCI-S or controls.

Longitudinally, we found evidence that mean adjusted hippocampal atrophy rates were higher in ε4+ in AD, MCI-P and MCI-S but not in controls. We also found evidence that mean adjusted hippocampal atrophy rates were higher in ε4+ in AD and MCI-P after adjusting for concurrent whole-brain atrophy rates. The difference in hippocampal atrophy rates in MCI-S was no longer significant after adjustment for concurrent brain atrophy rate.

Taken together these results demonstrate that ε4 carriers with a clinical diagnosis of AD or of progressive MCI have a different pattern of atrophy - disproportionately greater hippocampal loss - than non-carriers. Cross-sectional studies have shown reduced hippocampal volumes in ε4+ compared with ε4- in AD. However, without investigating longitudinal changes in hippocampal volume, it is not possible to tell whether these findings could be perhaps explained by developmental differences. Indeed, there is evidence that there are some developmental differences with one study reporting higher Mental Development Index scores in 24 month old babies who were ε4+ compared with those who were ε4− [34]. There are few studies in healthy young people comparing hippocampal volumes in ε4+ and ε4−. One study in a large cohort of adolescents reported no significant difference in hippocampal volumes between ε4+ and ε4− [35] whilst another smaller study in young adults reported significantly smaller hippocampi in ε4+ [36]. However, the study in adolescents did not adjust for head size whilst the study in young adults did, which makes comparisons between the studies difficult. Further studies would be required to understand the developmental differences between ε4+ and ε4−.

In older adults previous longitudinal studies have reported higher hippocampal rates in ε4+ compared with ε4−. However, higher rates of hippocampal atrophy in ε4+ could be potentially explained by higher rates of whole-brain atrophy (i.e. a more aggressive disease course with a more rapid loss of whole-brain tissue). In order to disentangle the effects of the ε4 allele on global and local hippocampal atrophy it is necessary to adjust hippocampal atrophy rates for global atrophy rates (whole-brain). In this study we found that hippocampal atrophy rates were still higher in ε4+ in AD and progressive MCI following adjustment for whole-brain atrophy rates. This suggests that higher hippocampal atrophy rates found in ε4+ are unlikely to be simply due to a more aggressive disease with faster disease progression (as measured by generalised brain tissue loss) alone. It may be that AD associated with the ε4 allele is a different anatomical disease to AD without this allele, which should be considered when assessing the effect of potentially disease modifying treatments.

Our finding of a lack of substantive differences between ε4+ and ε4− in hippocampal volume and atrophy rate in healthy control subjects is in agreement with some previous findings [15], [16], [21], [24]. Conversely, a number of previous studies have reported increased hippocampal atrophy rates for ε4+ compared with ε4− controls [11]–[13], [17], [19], [20], [25], [37], [38]. However, inconsistencies in findings between our study and that of some of the others may be due to different recruitment strategies: some studies had less stringent inclusion criteria than ADNI by including some MCI subjects with controls [17], [37]; some had a majority of subjects with a 1^st^ degree relative with a history of AD [12]. Differences in study design may also explain inconsistencies: some studies measured atrophy over a longer period, thus increasing the power with which to estimate differences in atrophy rates [13], [17], [38]. In the largest longitudinal study to date, with over 200 ε4 heterozygotes, no evidence of a difference in rates between heterozygotes and non-carriers was found [13], consistent with our findings.

Interestingly, different studies using subsets of the controls in the ADNI cohort have reported conflicting findings. Some reported significant evidence of an association between *APOE* genotype and bilateral hippocampal atrophy rate [11], [20]. One study that analysed the left and right sides separately reported a significantly higher rate of hippocampal atrophy on the right side hippocampus in ε4+ compared with ε4− [25] another reported a significantly higher atrophy rate in the left hippocampi in ε4+ compared to ε4− [19]. Others found no such association [16], [21]. Differences between findings of these studies and our own may be due to inclusion of ε2 carriers in most studies since ε2 carriers have shown lower hippocampal atrophy rates compared with non-carriers [26].

Reported results in MCI subjects are also mixed; a number of publications have shown a significantly greater hippocampal atrophy rate in ε4+ compared with ε4− [11], [16], [22], [25]. One study reported a significantly greater atrophy rate in the left hippocampus [19]. Conversely other studies reported no significant difference between ε4+ and ε4− in hippocampal atrophy rate in MCI [21], [24].

In the majority of the studies using data from ADNI an association has been found between ε4 carrier-status and higher hippocampal atrophy rates in MCI much like our own study. This is unsurprising in many ways since the MCI group has a high proportion of subjects who will progress to clinical AD; these subjects are more likely to be ε4+ and more likely to have increased hippocampal atrophy when compared with the MCI subjects who remain stable and may be less likely to have underlying AD pathology and less likely to be an ε4 carrier.

Other studies have examined hippocampal atrophy rates in MCI-S and MCI-P separately. One study, using voxel based morphometry (VBM) found increased hippocampal atrophy rates in MCI-P ε4+ compared with ε4- but not in MCI-S [22]. Another study, which used a number of hippocampal measures, found significantly higher rates in ε4+ in all measures in the MCI stable group [20]. In MCI-P they only found significantly increased loss of hippocampal grey matter (GM) density and GM volume in ε4+ but not hippocampal volume (as measured by FreeSurfer). We found no evidence of a difference in hippocampal atrophy rates in the MCI-S group after adjusting for concurrent whole-brain atrophy rate.

Our finding in AD of smaller hippocampi in ε4+ at baseline compared with ε4− is in keeping with a previous study which reported evidence of a negative association between ε4 dose and normalised hippocampal volume in AD subjects when adjusting for other covariates such as MMSE score [39]. Further, our longitudinal findings in AD of increased hippocampal atrophy rates in ε4+ compared with ε4− are in line with some previous studies [16], [18], [21]. Other studies report mixed or negative results for this comparison which may depend on the image analysis methodology: one study reported increased hippocampal GM atrophy in ε4+ but no significant increase in hippocampal atrophy (as measured with FreeSurfer) or GM density changes [20]; Others found no significant difference in hippocampal loss rates between ε4+ and ε4− in AD [11], [15].

A strength of our study was the relatively large number of subjects with data from multiple time-points (up to 36 months from baseline). ADNI has the advantage of being a prospective study with standardised follow-up times and high quality MRI imaging. We used the MAPS hippocampal segmentation technique which has been shown to have good accuracy when compared with manual segmentations [31]. In addition, the analysis method has the advantage of a robust and direct longitudinal measure of hippocampal and whole brain change, the BSI.

This study also has a number of limitations. First the ADNI clinical diagnoses have not been pathologically confirmed and it may be that some AD diagnoses will prove to be caused by non-AD pathology at autopsy. Secondly, since our segmentation method (hippocampal-MAPS) excludes the hippocampal tail, and it is possible that atrophy rates differ across hippocampal sub-regions, we could be potentially missing early changes in control subjects positive for the ε4 allele and including this region in all subject groups may change the results. Thirdly, the longitudinal model assumes that the missing observations were missing at random, an assumption which cannot be empirically verified. Finally, we excluded subjects with an ε2 allele since we did not want this to confound our results. It would be of particular interest to investigate hippocampal atrophy rates in ε2/ε4 subjects as compared with other genotypes to evaluate whether ε2 or ε4 has greater influence on rates; however this genotype was rare in this dataset (only 3 controls, 2 MCI-S, 5 MCI-P and 2 ADs had the ε2/ε4 genotype).

In summary, we have investigated the association of hippocampal volume and hippocampal atrophy rate with *APOE* genotype, while adjusting for age, gender, cognitive impairment (MMSE score), baseline atrophy level (brain-to-TIV ratio) and head size as well as interval between scans in the longitudinal analysis. We found evidence that within the AD group ε4+ had lower mean adjusted hippocampal volumes at baseline compared with ε4−. We found evidence that AD, MCI-P and MCI-S ε4+ had higher mean adjusted hippocampal atrophy rates compared with ε4− and furthermore that in AD and MCI-P ε4 carriers still showed higher mean adjusted hippocampal atrophy rates after adjustment for concurrent whole-brain atrophy rates (which, to our knowledge, has not be previously shown). Higher atrophy rates in ε4+ suggest that the patterns of atrophy are not merely manifestations of developmental differences according to genotype. Our results thus support the hypothesis that in AD the ε4 allele influences disease phenotype with greater hippocampal involvement compared with non-carriers.

## Supporting Information

Appendix S1 **List of included subjects.**
(XLSX)

Appendix S2 **Statistical models.**
(DOC)
