# Correction: *APOE* ε4 Is Associated with Disproportionate Progressive Hippocampal Atrophy in AD

**DOI:** 10.1371/journal.pone.0104482

**Published:** 2014-07-29

**Authors:** The PLOS ONE Staff

## Notice of Republication

This article was republished on July 7, 2014, to correct an error in the byline; the publisher apologizes for the error. In addition, Figures 1-6 were resized. Please download this article again to view the correct version. The originally published, uncorrected article and the republished, corrected article are provided here for reference.

## Supporting Information

File S1 Originally published, uncorrected article.
(PDF)

File S2 Republished, corrected article.
(PDF)
