# Correction: *APOE* ε4 Is Associated with Disproportionate Progressive Hippocampal Atrophy in AD

**DOI:** 10.1371/journal.pone.0110346

**Published:** 2014-09-30

**Authors:** The PLOS ONE Staff

Figure 3 is incorrect. The first p-value should be above the second column, not the first. Please see the correct figure below.

**Figure 3:**
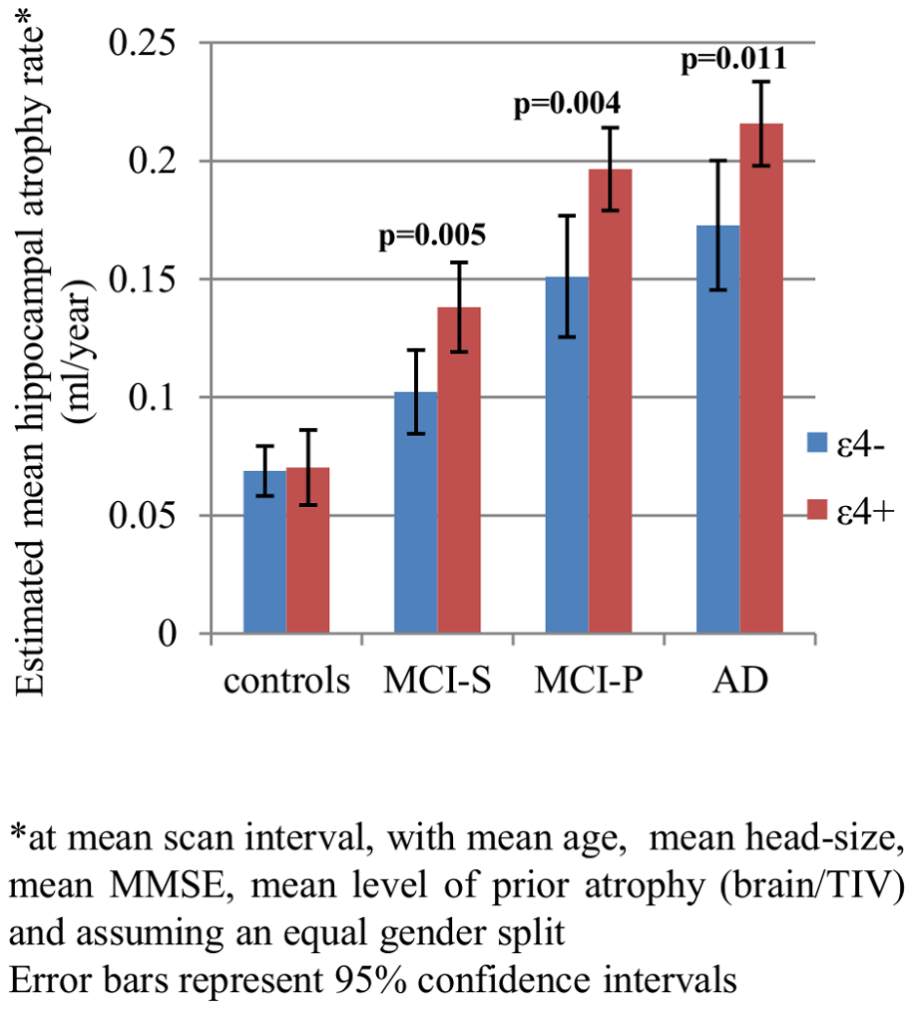
Effect of *APOE* ε4 on hippocampal atrophy rates.*
