# Supplementary material for: APOE ε4 Is Associated with Disproportionate Progressive Hippocampal Atrophy in AD
Source: PLoS One. 2014 May 30;9(5):e97608. doi: 10.1371/journal.pone.0097608 (PMC4039513; doi:10.1371/journal.pone.0097608)
Supplement: Appendix S2 — Statistical models. (DOC) [file pone.0097608.s002.doc]

Appendix 2: Statistical models

*Cross-sectional analysis*

For the analysis examining the influence of the APOE ε4 allele on baseline hippocampal volumes, the following linear regression model was used:

|  | $\begin{matrix} Y_{i}\text{=}\beta_{0}+\beta_{1}e4carrier_{i}+\beta_{2}age_{i}+\beta_{3}female{}_{i}+\beta_{4}mmse_{i} \\ +\beta_{5}brainTIVratio_{i} +\beta_{0}TIV_{i}+\varepsilon_{i} \end{matrix}$ | 1.1 |
| --- | --- | --- |

and

|  | $\varepsilon_{i}\sim N(0,\sigma_{\varepsilon1}^{2})$ | 1.2 |
| --- | --- | --- |

$Y_{i}$ is the hippocampal volume for subject *i*, *e4carrier* is a categorical variable representing the APOE ε4 carrier status (0 if non-carrier, 1 if carrier), *age* is the mean centred age, *female* is a categorical variable for gender (0 if male, 1 if female), *mmse* is the mean centred MMSE score, *brainTIVratio* is the mean centred brain to total intracranial volume ratio, *TIV* is the mean centred total intracranial volume, $\varepsilon_{i}$is the error term, $\beta_{0}$is the baseline hippocampal volume in non ε4-carriers, $\beta_{1}$is the difference in volume between non-carriers and carriers and $\beta_{2},\beta_{3},...,\beta_{6}$are the fixed effects coefficients corresponding to the other covariates.

*Longitudinal analysis*

For the analysis examining the influence of the APOE ε4 allele on rates of hippocampal and whole brain atrophy the following joint mixed model was used:

|  | $\begin{matrix} Y_{ij}^{(1)}=(\beta_{0}^{(1)}+\beta_{1}^{(1)}e4carrier_{i}+\beta_{2}^{(1)}age_{i}+\beta_{3}^{(1)}female_{i}+\beta_{4}^{(1)}mmse_{i} \\ +\beta_{5}^{(1)}brainTIVratio_{i} +\beta_{6}^{(1)}TIV_{i}+b_{i}^{(1)})t_{ij}+\varepsilon_{ij}^{(1)} \end{matrix}$ | 2.1 |
| --- | --- | --- |
|  | $\begin{matrix} Y_{ij}^{(2)}=(\beta_{0}^{(2)}+\beta_{1}^{(2)}e4carrier_{i}+\beta_{2}^{(2)}age_{i}+\beta_{3}^{(2)}female_{i}+\beta_{4}^{(2)}mmse_{i} \\ +\beta_{5}^{(2)}brainTIVratio_{i} +\beta_{6}^{(2)}TIV_{i}+b_{i}^{(2)})t_{ij}+\varepsilon_{ij}^{(2)} \end{matrix}$ | 2.2 |

Here $Y_{ij}^{(1)}$ denotes the jth measurement of brain loss between baseline and time $t_{ij}$ for subject i, and $Y_{ij}^{(2)}$ the corresponding measure of hippocampal loss. $\beta_{0}^{\left(1\right)}$is the mean adjusted brain atrophy rate in non ε4 -carrier males with mean age, mean mmse score, mean brain-to-TIV ratio and mean headsize, $\beta_{1}^{\left(1\right)}$ is the difference in mean adjusted brain atrophy rate between ε4 carriers and non-carriers, $\beta_{2}^{\left(1\right)},\beta_{3}^{\left(1\right)},...,\beta_{6}^{\left(1\right)}$ are the fixed effect coefficients for the other covariates (age, gender, MMSE score, brain-to-TIV ratio and TIV) for brain loss and $b_{i}^{(1)}$ is the random effect slope for subject i, likewise $\beta_{0}^{\left(2\right)}$is the mean adjusted hippocampal atrophy rate in non ε4-carrier males with mean age, mean mmse score, mean brain-to-TIV ratio and mean headsize, $\beta_{1}^{\left(2\right)}$ is the difference in mean adjusted hippocampal atrophy rate between ε4 carriers and non-carriers, $\beta_{2}^{\left(2\right)},\beta_{3}^{\left(2\right)},...,\beta_{6}^{\left(2\right)}$are the fixed effect coefficients for hippocampal loss, and is the random slope for subject i.

We assumed the random slopes satisfy

|  | $\left(\begin{matrix} b_{i}^{(1)} \\ b_{i}^{(2)} \end{matrix}\right)\sim N\left(\left(\begin{matrix} 0 \\ 0 \end{matrix}\right),\left(\begin{matrix} \sigma_{b1}^{(2)} & \sigma_{b1b2} \\ \sigma_{b1b2} & \sigma_{b2}^{(2)} \end{matrix}\right)\right)$ | 2.3 |
| --- | --- | --- |

and

|  | $\left(\begin{matrix} \varepsilon_{i}^{(1)} \\ \varepsilon_{i}^{(2)} \end{matrix}\right)\sim N\left(\left(\begin{matrix} 0 \\ 0 \end{matrix}\right),\left(\begin{matrix} \sigma_{\varepsilon1}^{(2)} & \sigma_{\varepsilon1\varepsilon2} \\ \sigma_{\varepsilon1\varepsilon2} & \sigma_{\varepsilon2}^{(2)} \end{matrix}\right)\right)$ | 2.4 |
| --- | --- | --- |

The covariance allows the residual errors within visits to be correlated for the two measures and the covariance allows the random slopes to be correlated. This enables us to adjust the difference in hippocampal atrophy rates between ε4 carriers and non-carriers for concurrent whole brain atrophy rate.
